# Evaluation of the Bond Strengths between Dental Porcelain and Cobalt-Chromium Metal Frameworks Manufactured with Different Techniques after the Thermal Aging Process

**DOI:** 10.1155/2020/9315236

**Published:** 2020-04-29

**Authors:** Elif Ece Yoldan, Nurullah Türker, Ulviye Ş. Büyükkaplan, Mehmet M. Özarslan, Recep Karalı, Ahmet T. Deniz

**Affiliations:** Akdeniz University Faculty of Dentistry, Department of Prosthodontics, Antalya 07058, Turkey

## Abstract

**Purpose:**

The present study is aimed at examining the bond strength of cobalt-chromium (Co-Cr) metal frameworks, prepared through different techniques, to a single type of low-temperature porcelain system after the thermal aging process.

**Methods:**

A hundred and twenty Co-Cr alloy framework specimens were prepared using conventional casting, CAD/CAM, and two commercially different laser sintering devices, and dental porcelain was applied to the specimens. A single type of dental porcelain (Kuraray Noritake Dental Inc., Tokyo, Japan) was applied to the specimens. After the subgroups were determined, half of the specimens were subjected to a thermal aging process. Bond strength of specimens was evaluated using a 3-point bending test. The surfaces of the fractured specimens were evaluated using a stereomicroscope. The metal-porcelain bonding area of samples randomly selected from 8 groups has been examined with SEM under ×1000 magnifications. Normality distribution of obtained data was examined using by a Kolmogorov-Smirnov test. The obtained data of the present study was statistically analyzed with a statistical package program (SPSS for Windows 22.0, Chicago, IL, USA).

**Results:**

There was a statistically significant difference between CAD/CAM and the other three methods, and the bonding value of the CAD/CAM group was the highest among the groups. Besides, the bond strength between dental porcelain and 4 differently produced metal frameworks was high enough to surpass the acceptable threshold (>25 MPa) according to the ISO 9693. There was no statistically significant difference between thermal aging applied and nonapplied groups.

**Conclusions:**

Based on this study, it could be shown that the metal-ceramic bond strength is dependent on the manufacturing method used, but it is independent of the thermal aging application. It was found that the bond strength values of all samples with and without thermal aging application exceeded the minimum acceptable value of 25 MPa recommended by the ISO 9693.

## 1. Introduction

Although there is a great demand for metal-free restorations in contemporary dentistry, successful use of porcelain fused to metal restorations is still an important part of dental treatments today. Combining the aesthetic properties of ceramic and the durability of metal, the fact that mostly desirable results are achieved in eliminating functional and aesthetic losses of a patient, satisfying physical properties, and lower costs compared to ceramic restorations without metal reinforcement cause porcelain fused to metal restorations to still be valuable for clinicians despite advanced metal-free restorations [1–3].

In the production of metal frameworks of porcelain fused to metal restorations, cobalt-chromium (Co-Cr) alloys, which achieve a high rate of clinical success and do not contain Ni allergen, are commonly preferred [4, 5]. Errors by the technical staff at the laboratory stage, difficulties faced due to the impressions taken from the patient and the attained plaster model at the clinic, lost wax, and the conventional casting method may pose an obstacle for achieving ideal results in the preparation of metal frameworks [6, 7]. Today, with technological developments, systems with computer-assisted design-computer-aided manufacturing (CAD/CAM) technologies have been developed for fast production of fixed restorations, so that negative aspects of the conventional casting method can be eliminated [8, 9]. Frameworks can be produced either with a CAD/CAM system based on the principle of milling from prefabricated blocks or with a CAD/CAM system based on the principle of adding materials layer by layer by using these systems [10], which have advantages such as lowering the costs and avoiding any problems during the casting process compared with the conventional casting method [8, 11, 12]. The facts that drills used in the metal framework production process with the milling technology is exposed to overabrasion, that the system cannot achieve the desired savings on time, that residues of milled prefabricated blocks are excessive, and that production of multiple complex structural restorations simultaneously is highly challenging result in a recent concentration on the use of “Rapid Prototype Production Techniques” in prosthetic dentistry [13, 14].

Most of the recent studies about the success of the porcelain fused to metal restorations have focused on the metal porcelain bonding [4, 15–18]. Despite the fact that the literature mostly includes studies evaluating the metal-ceramic bond strengths of Co-Cr metal substructures prepared through the latest production methods and conventional casting methods [3, 7, 10], there are a few studies in which in vitro tests, which imitate intraoral temperature changes, are included [19]. Therefore, the purpose of the present study was to examine the bond strength of Co-Cr metal frameworks, prepared through different production techniques, to a single type of low-temperature porcelain system (Kuraray Noritake Dental Inc., Tokyo, Japan) after the thermal aging process and elucidate the effects of different metal substructure preparation techniques on the bonding established between the metal framework and dental porcelain. The null hypothesis in the present study was that thermal aging and production methods have no effects on the bond strength between differently produced metal frameworks and dental porcelain.

## 2. Materials and Methods

**Figure 1:**
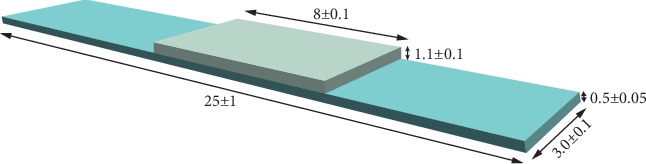
The illustration of a specimen prepared according to ISO 9693 standard for the present study.

In the present study, Co-Cr frameworks manufactured by using three methods with two different sintering devices were evaluated. For this purpose, a hundred and twenty Co-Cr alloy frameworks with the dimensions of 25 × 3 × 0.5 mm stated according to ISO 9693 standard specimens were prepared using conventional casting, CAD/CAM, and two commercially different laser sintering devices. A single type of dental porcelain with the dimensions of 8×3×1.1 mm was applied to the specimens, and after the subgroups were determined, half of the specimens were subjected to the thermal aging process. An illustration of a specimen used in the present study is shown in Figure 1. Production methods and the material content of Co-Cr metal frameworks are shown in Table 1.

### 2.1. Samples Prepared with Conventional Casting Techniques

Wax models prepared to be used in the metal alloy specimens produced with the conventional casting technique (Mayka Expert 7,5, Picasoft, Vierzon, France) were designed, and 30 pieces of standard wax models were prepared with the CAD/CAM technology using wax blocks with suitable shapes for the casting system (Alma-Dent Co. Ltd., Izmir, Turkey). Specimens included in the revetment (Granitite Presto Vest II, Salient Dr. Bohme & Shops GmbH, Goslar, Germany) were cast with an induction heating and centrifuge casting furnace (Microtel Dental, Ankara, Turkey), using a new Co-Cr metal alloy tablet for each of the specimens (KERA® C, Eisenbacher Dentalwaren ED GmbH, Wörth am Main, Germany) for the casting process.

### 2.2. Samples Prepared with the CAD/CAM Method

A 3D virtual model of the metal specimens prepared with the CAD/CAM method was designed with a CAD software program (Mayka Expert 7,5, Picasoft, Vierzon, France). Afterwards, in order to proceed with the production stage, the model was exported to the CAM unit of the system in STL format, and the Co-Cr-based disk-shaped metal block (Magnum Lusens Co-Cr, Mesa di Sala Giacomo & C. S.N.C., Brescia Province, Italy) was prepared with the CAM device (Yena D40, Yenadent, İstanbul, Turkey) that can abrade on five axes.

### 2.3. Samples of the Selective Laser Sintering EOS System

In order that Co-Cr-based metal framework specimens of the first group under the selective laser sintering system could be prepared, Cr-Co alloy powder (EOS CobaltChrome SP2, EOS GmbH, Krailling, Germany) with a thickness of 30 *μ*m was laid on the stainless steel production platform using cylinders of a selective laser sintering system (Eosint M270, EOS GmbH, Krailling, Germany). The layered metal structure was solidified by melting and binding the alloy powder used during the production of metal substructure samples of the system (Eosint M270, EOS GmbH, Krailling, Germany) using a laser beam in accordance with the virtual design. Set-up parameters of the laser system and firing schedule of each metal framework after laser production are shown in Tables 2 and 3, respectively.

### 2.4. Samples of the Selective Laser Sintering Concept System

Upon completing the virtual designs of second group metal substructure samples of the selective laser sintering system, CAD data was sent to the head office (4C Engineering Co. Ltd., Istanbul, Turkey), so that production could be carried out. Production of 30 samples of 30 *μ*m thickness was carried out through melting and binding the Co-Cr-based metal powder (Remanium® star CL, Dentaurum GmbH & Co. KG, Ispringen, Germany**)** in a selective laser sintering device (Concept Laser Mlab, Concept Laser GmbH, Lichtenfels, Germany) layer by layer in accordance with the virtual design. Set-up parameters of the laser system and firing schedule of each metal framework after laser production are shown in Tables 2 and 3, respectively.

### 2.5. Application of Dental Porcelain

Dimensions of metal specimens prepared for all groups have been checked with a caliper (Dial Caliper, Starrett Company, Massachusetts, USA) and leveled, and the samples have been made ready for the oxidation process. Before the oxidation process, 120 pieces of metal substructure samples had been kept in an ultrasonic cleaner (BioSonic UC50, Coltène/Whaledent GmbH&Co. KG, Langenau, Germany) with distilled water for 10 minutes and then in ethyl alcohol for another 10 minutes so that the residues on the surface could be cleaned. After the oxidation process, samples have been blasted with Al_2_O_3_ particles (from a 1 cm distance, for 15 seconds, at 45° angle, under 2 atm pressure) of 125 *μ*m in diameter. The center 8 mm parts of the samples on one surface were measured in order to ensure that, for ceramic application, the opaque layer can be applied at the same place and with the same dimensions on each sample. NP Bonder of Paste Opaque is used for the porcelain as a recommendation of the manufacturer. Opaque (two-stage) and dentin ceramic powder (with the layering technique) of a porcelain system developed to be used with metal alloys have been applied, respectively, on the bordered areas on each sample. Thickness of powder opaque first, powder opaque second, and powder dentin ceramic was 0.1 mm, 0.1 mm, and 0.9-1 mm, respectively. The ceramic firing process of the samples was performed in a ceramic furnace (Tegra MP2100, Teknik Dental, İstanbul, Turkey) according to the instructions of the manufacturing company (Table 4). Dimensions were checked with a micrometer (Dial Caliper, Starrett Company, Massachusetts, USA) in order to ensure that the total porcelain dimensions of all fired samples were 8 × 3 × 1.1 mm. The dimensions of porcelain and metal of a completed specimen are also shown in Figure 1. The porcelain Young's modulus and thermal expansion coefficient (WAK) values were 104 GPa and 12.4 × 10^−6^, respectively.

### 2.6. Thermal Aging Procedure

Studies show that temperature values inside the mouth vary between 5°C and 55°C depending on the nutritional conditions [20, 21]. Accordingly, these conditions in the mouth were tried to be imitated with the thermal cycle process. Half of all experimental group samples were put in thermal cycling upon completion of the dental porcelain application; the cycle was completed before the three-point bending test in a thermal cycling device (MOD-Dental Thermal Cycling, Esetron Mechatronics Engineering and Electronics Industry, Ankara, Turkey) set to ensure that retention time in tap water tanks at +5°C and +55°C, respectively, is 30 seconds, and the transition time between two tanks is 15 seconds upon performing 5000 cycles. In order to avoid any sample confusion between the groups during the cycle, the samples of the four groups were placed in the sample basket of the device in a mesh bag.

### 2.7. Evaluation of the Metal-Ceramic Bond

Bond strengths of samples of 8 groups with and without exposure to the thermal aging process were evaluated with the 3-point bending test [22], using a desktop universal testing device (Shimadzu Scientific Instruments Inc., Kyoto, Japan) in accordance with the ISO 9693 standard. Samples with ceramic superstructures facing down were placed on the device, which was adjusted so that the distance between two supports was 20 mm. The force applying bit was adjusted to proceed at the rate of 1.0 mm/min vertically to the center of the metal surface on the samples until the breaking moment of the metal-ceramic interface bond. The force (N) that caused a bond failure on the metal-ceramic interface was recorded by the software (Trapezium X Material Testing Operation Software, Shimadzu, Kyoto, Japan) on the computer connected to the universal testing device. In the present study, the *E*-modulus of Co-Cr compositions for conventional casting, CAD/CAM, Concept Laser, and EOS Laser sintering was 170, 194, 230, and 200 GPa, respectively.

Metal-porcelain bond strengths (MPa) of the groups were attained through using the force values that initiated bonding failure in the formula of the “*τb* = *k* × *F*” equation (Table 3).

### 2.8. Stereomicroscope and Scanning Electron Microscopy (SEM) Analysis

After the 3-point bending test, the surfaces of the fractured specimens were evaluated using a stereomicroscope (Stemi SV 11 APO, Carl Zeiss, Oberkochen, Germany). Failure types of specimens were recorded at this stage. The metal-porcelain bonding area of samples randomly selected from 8 groups has been examined with SEM (ZEISS LEO 1430, Carl Zeiss, Oberkochen, Germany) under ×1000 magnifications. Before evaluation of SEM images, specimen surfaces were sputtered with an Au-layer (Polaron SC 7620 Sputter Coater, Quorum Technologies Ltd., Kent, GB).

### 2.9. Statistical Analysis

In the present study, Student's *t*-test was used in independent groups, and one-way analysis of variance (ANOVA) was adopted for comparison of measurement values of more than two groups. Probability values of *p* < 0.05 were considered to be statistically significant.

## 3. Results

**Figure 2:**
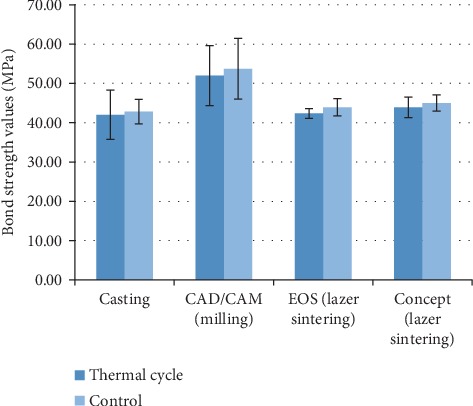
The results of bond strengths (MPa) based on different production methods between the groups with and without thermal aging application.

**Figure 3:**
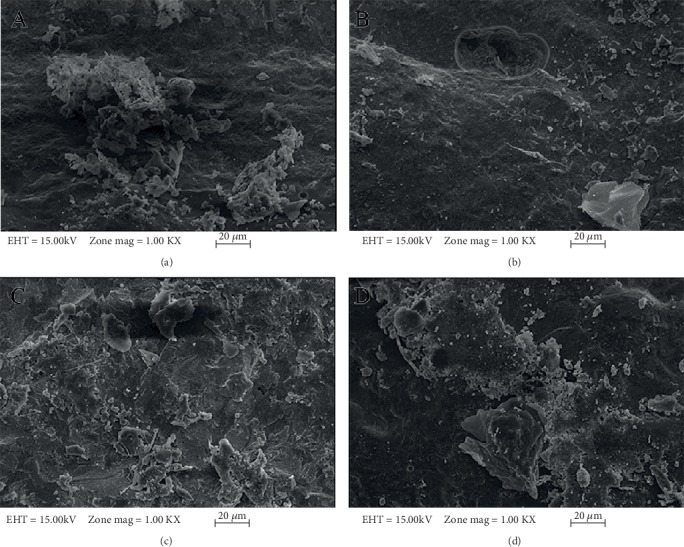
SEM analysis of samples, each selected from one of the four groups without thermal cycling application, under ×1000 magnification: (a) conventional casting method; (b) CAD/CAM system; (c) EOS Laser sintering; (d) Concept Laser sintering.

**Figure 4:**
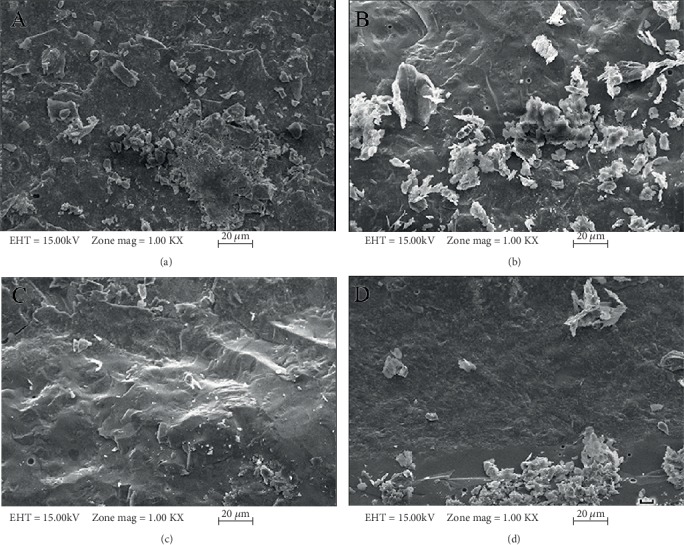
SEM analysis of samples, each selected from one of the four groups with thermal cycling application, under ×1000 magnification: (a) conventional casting method; (b) CAD/CAM system; (c) EOS Laser sintering; (d) Concept Laser sintering.

Figure 2 shows the bond strength values of all groups in the present study. According to the results of descriptive statistics for the bond strengths (MPa) and the ANOVA test results for metal substructure samples prepared in the study based on different production techniques, there was a statistically significant difference between the bond strength values upon the comparison of the strength values (*p* < 0.01). The Duncan multiple comparison test was applied in order to determine the production method resulting in such difference. There was a statistically significant bond strength value difference between the CAD/CAM group and the other 3 groups (Table 5). However, there was no significant difference between the conventional casting and commercially different 2 laser sintering groups. Descriptive statistics for porcelain bond strengths (MPa) and *t*-test results of thermal aging applied and nonapplied groups in the study showed that there was no statistically significant difference between the metal-ceramic bonding values of groups exposed to the thermal aging and groups not exposed to the thermal aging process (Table 6). In most of the examples, a mixed failure type was observed, and none of the examples showed cohesive failure. There was no effect of thermal cycle application on failure types (Tables 7 and 8). Figures 3(a)–3(d) and Figures 4(a)–4(d) show SEM analysis of samples under ×1000 magnification, each selected from one of the four groups without thermal cycling application and with thermal cycling application, respectively. In all the SEM images, the presence of porcelain remains on the metal framework can be seen. Unlike other images, Figures 3(b) and 4(d) also contain deep grooves.

## 4. Discussion

The basis of the long-term success of porcelain fused to metal restorations was the formation of a bond between the metal and porcelain that is strong enough to withstand the stresses created in the oral cavity. Results of this study, in which the effects of both different production techniques and thermal aging process, imitating the intraoral temperature changes, on the bond strength of Co-Cr metal substructures to the single type low-temperature porcelain system were evaluated, indicating that the bond values of CAD/CAM group samples with and without thermal cycling application are significantly higher than average bond strength values of other groups.

This study is unique in that it evaluates the effect of different production methods on metal-ceramic connection by considering intraoral temperature changes. There is no consensus on the number of thermal cycles and immersion time in the literature. The thermal cycle protocol published for dental materials recommends that thermal cycle tests be performed in the lowest 5°C and the highest 55°C interval, with an average retention time of 30 seconds [20, 21]. In the present study, 5000 cycles and 5°C to 55°C applications were carried out in accordance with ISO/TS 11405 recommendation [23]. While thermal cycling application did not cause a significant difference in bond strength values, samples of all groups not having undergone the thermal cycling application had higher average binding values compared to sample groups having undergone the thermal cycling application. This effect of the thermal cycle has also been observed in other studies [18, 24]. The results of this study indicate that bond strength values before and after thermal cycling application are independent of production techniques. Similarly, with the results of many previous studies, mixed type failure has been the most frequently encountered failure in the present study [2, 4]. According to the results of this study, no direct relationship has been established between the bonding failure type and the bond strength. However, according to the results of the study, as no statistically significant difference was found between bond strength values of the CAD/CAM (milling/drilling) group that did not undergo the thermal cycling application and the other 3 groups that did not undergo the thermal cycling application, the null hypothesis was rejected. Furthermore, as there was no statistically significant difference between the groups with and without thermal cycling application, the null hypothesis was accepted in that case.

In a similar study, Han et al. reported that the bond strength values of samples produced with the Concept Laser were higher than those produced with the traditional casting method and CAD\CAM [16]. The present study is different from the study of Han et al. in terms of the presence of the thermal cycle application and the content of the metal alloys used. The reason for the difference in findings can be attributed to the difference in the content of the metal alloy. The findings obtained by Zhou et al. in their study by adding lanthanum in different Co-Cr alloys support this view [25]. A related study showed a statistically significant difference between the groups in terms of bond strength. In another study conducted by Zhou et al., the bond strength values were compared in the samples produced with the traditional casting method and Concept Laser, and it was reported that the bond strength values were higher in the samples produced with the traditional casting method [26]. For this reason, when comparing the studies that examine the metal framework materials produced using different production techniques from various aspects, the details of the production method, the content of the metal powder used, and the nature of the thermal cycle applications should be carefully evaluated.

Although laser sintering methods with different application parameters were used in our study, no significant difference was observed between the bond strength values. The results of a study conducted by Tulga and Küçükekenci support this finding [27]. Researchers compared shear bond strength values of composite resin material connected on framework produced with EOS and Concept Lasers. In the study, no significant difference was observed between these two production methods.

Stawarczyk et al. [28], with three different Co-Cr alloy frameworks (Ceramill Sintron; Milleme, Ceramill NP L; Laser, Girobond NB; casting), which they obtain by casting, milling, and laser sintering and subjecting to thermal cycle application, investigated the bond strengths of ceramics (Creation, VITA VM 13, Reflex). As a result of the study, the samples have similar bond strength values according to the construction methods. However, considering the connection strength between ceramic types and metal frameworks, they found that the Creation brand ceramic has better bond strength than VITA VM 13 and Reflex. In our study, one type of porcelain was used and a significant difference was observed between the production techniques in terms of bond strength. Further studies should be carried out in which different metal substructure production techniques evaluate the bond strength with different ceramic types.

Furthermore, Li et al. [3] found out in their studies, in which the metal-ceramic bonding area is examined through SEM/EDS analysis, that metal frameworks obtained through different production methods have similar surface morphologies as a result of the blasting process. The literature includes studies in which conditions that can affect bond strength, such as surface preparations, changes in ceramic firing conditions, and preferred alloy type, are evaluated. In the present study, in order to standardize surface properties, all samples were blasted with Al_2_O_3_ particles 125 *μ*m in diameter under 2 atm pressure, and the same firing conditions were provided with a single type of ceramic material in accordance with the specifications of the manufacturer. The aim was to avoid variations caused by different surface and firing processes, which could influence the absolute effect of different production methods and thermal cycling on bond strength.

Different components in alloys may cause variations in mechanical properties of the alloys and porcelain bond strength. Ekren et al. [29] in their study, in which a single type of dental porcelain was applied to Co-Cr metal substructures prepared through two different direct metal laser production methods (DMLS and DMLM), have evaluated the effect of operation principle, layer thickness, and alloy powder content differences among devices used in production methods on metal porcelain bond strength. Their study found that, rather than differences in layer thicknesses, variations in operating principles of devices used in production methods and differences in the contents of alloy powders significantly affect metal to porcelain bonding. In our study, contrary to this result, while no statistically significant difference was observed between metal-ceramic bond strengths of metal alloys produced with laser sintering devices from two different manufacturers and Co-Cr alloy powder contents, it was determined that the average bond strength values of metal substructures produced with the Concept Laser company's device were higher than the bond strength values of those produced with the EOS company's laser sintering device.

Despite the fact that an ideal testing method is not agreed on [17], the three-point bending test was also preferred in our study for determining the metal to porcelain bond strength because samples used in the three-point bending test can be prepared easily according to DIN and ISO standards, and it is recommended by the American Dental Association Council on Dental Materials, Instruments, and Equipment. According to the results of the present study, it was observed that all samples with and without the thermal cycling process were higher than 25 MPa, which is the lowest metal-ceramic bonding value according to the ISO 9693 standard.

## 5. Conclusions

The following was concluded in this in vitro study:

1. It was determined that the bond strength of samples obtained through the CAD/CAM method was higher than bond strength values obtained through conventional casting and using two different laser sintering systems
2. In the present study, while no difference was found between bond strength (MPa) values of the groups established based on the thermal aging application on the prepared samples, it was determined that samples subjected to thermal aging had lower bond strength values compared to samples not subjected to thermal aging. It was observed according to the three-point bending test results that bond strength values of samples in all groups exceed 25 MPa, which is the clinically acceptable limit

## Figures and Tables

**Table 1:** Production methods and the material content.

Manufacturing method	Material	Composition	Manufacturer	Device
Traditional casting	KERA® C, cobalt-based type V metal alloy	60% Co, 24.5% Cr, 9 W%, 1.1% Mo, 0.9 Si%	Eisenbacher Dentalwaren ED GmbH	Mikrotek Dental
CAD/CAM (milling)	Magnum Lusens Co-Cr	65% Co, 29% Cr, 2% Nb, 2% W	Mesa di Sala Giacomo & C. S.N.C.	Yena D40
Laser sintering	EOS CobaltChrome SP2	63.8% Co, 24.7% Cr, 5.1% Mo, 5.4% W, 0.5% Fe	EOS GmbH Electro Optical System	Eosint M270
Laser sintering	Remanium® star CL	60.5% Co, 28% Cr, 9% W, 1.5% Si	Dentaurum GmbH & Co. KG	Concept Laser Mlab

**Table 2:** EOS Laser and Concept Laser processing parameters.

	EOS Laser	Concept Laser
Environment	Argon atmosphere	Argon atmosphere
Scan speed	7 m/s	7 m/s
Focus diameter	40 *μ*m	40 *μ*m
Lamination thickness	30 *μ*m	25 *μ*m
Yb-fiber laser power	200 W	100 W
Heating-cooling procedure	Different	Different

**Table 3:** Furnace conditions after laser production.

EOS Laser		Concept Laser	
Room temp to 450°C	60 min	Room temp to 400°C	60 min
450°C	45 min	400°C	60 min
450°C to 750°C	45 min	400°C to 1150°C	60 min
750°C	60 min	1150°C	60 min
300°C	Extraction	300°C	Extraction

**Table 4:** Firing schedule of each porcelain layer.

	High temp (°C)	Predrying temp (°C)	Dry-out time (min.)	Heat rate (°C/min.)	Vacuum time (min)	Start vacuum (°C)	Release vacuum (°C)
Powder opaque first	980	500	8	65	1	500	80
Powder opaque second	980	500	8	65	1	500	980
Powder dentin ceramic	935	600	10	45	0	600	925

**Table 5:** Descriptive statistics and one-way ANOVA test result of binding strengths (MPa) based on different production methods.

	*N*	Mean (SD)	Min	Max	*p* value
Casting	30	42.41 (4.87)	31.98	55.03	
CAD/CAM (milling)	30	52.86 (7.60)	37.01	68.11	0.0001
EOS (laser sintering)	30	43.15 (1.92)	40.06	46.92	
Concept (laser sintering)	30	44.46 (2.38)	39.23	49.05	

SD: standard deviation; Min: minimum; Max: maximum.

**Table 6:** Descriptive statistics and *t*-test result of porcelain binding strengths (MPa) between groups with and without thermal aging application.

		*N*	Mean (SD)	Min	Max	*p* value
Casting	Thermal cycle	15	42.02 (6.26)	31.98	55.03	0.6679
Casting	Control	15	42.81 (3.11)	36.04	46.13	
CAD/CAM (milling)	Thermal cycle	15	51.99 (7.64)	37.01	65.58	0.5447
CAD/CAM (milling)	Control	15	53.72 (7.74)	40.57	68.11	
EOS (laser sintering)	Thermal cycle	15	42.36 (1.23)	40.06	44.22	0.0608
EOS (laser sintering)	Control	15	43.94 (2.19)	40.27	46.92	
Concept (laser sintering)	Thermal cycle	15	43.91 (2.63)	39.23	48.56	0.2109
Concept (laser sintering)	Control	15	45.01 (2.05)	41.03	49.05	

SD: standard deviation; Min: minimum; Max: maximum.

**Table 7:** Distribution of failure types in samples without thermal cycling.

		Failure type	
	Cohesive	Adhesive	Mixed
Casting	0	2	13
CAD/CAM (milling)	0	1	14
EOS (laser sintering)	0	4	11
Concept (laser sintering)	0	3	12

**Table 8:** Distribution of failure types in samples with thermal cycling.

		Failure type	
	Cohesive	Adhesive	Mixed
Casting	0	3	11
CAD/CAM (milling)	0	3	12
EOS (laser sintering)	0	5	10
Concept (laser sintering)	0	4	10

## Data Availability

The data used to support the findings of this study are included within the article.
